# Epidemiology of Bacterial Meningitis in the Lombardy Region, Italy, from 2014 to 2024: An Observational, Retrospective Study

**DOI:** 10.3390/microorganisms13081733

**Published:** 2025-07-24

**Authors:** Maria Francesca Liporace, Federica Salari, Beatrice Silvia Orena, Michela Piccoli, Elena Tomassini, Luigi Vezzosi, Gabriele Del Castillo, Laura Daprai, Danilo Cereda, Claudia Alteri, Annapaola Callegaro

**Affiliations:** 1 Microbiology and Virology Unit, Fondazione IRCCS Ca’ Granda Ospedale Maggiore Policlinico, 20122 Milan, Italy; 2 Residency in Microbiology and Virology School, University of Milan, 20122 Milan, Italy; 3 Directorate General for Health, Lombardy Region, 20124 Milan, Italy; 4 Department of Oncology and Hemato-Oncology, University of Milan, 20122 Milan, Italy

**Keywords:** bacterial meningitis, vaccine-preventable diseases, surveillance, *Haemophilus influenzae*, *Neisseria meningitidis*, *Streptococcus pneumoniae*

## Abstract

Bacterial meningitis remains a critical public health issue globally due to its high morbidity and mortality. Understanding regional epidemiological trends is essential to inform vaccination strategies and public health interventions. This observational, retrospective study analyzed cerebrospinal fluid (CSF) isolates collected from 731 confirmed cases of bacterial meningitis between 2014 and 2024 in Lombardy, Italy. Pathogen identification and serotyping of *Streptococcus pneumoniae* (SP), *Neisseria meningitidis* (NM), and *Haemophilus influenzae* (HI) were conducted using culture-based and molecular techniques. Trends were assessed across age groups and time using Kruskal–Wallis and chi-square tests. Results: SP was the predominant pathogen (78.4%), followed by NM (13.0%) and HI (8.6%). Significant temporal variation was observed for SP and NM, while HI trends remained stable. The impact of COVID-19-related restrictions was evident in a reduction in cases during 2020–2021. SP serotypes 3 and 8, HI non-typeable strains, and NM serogroup B were most frequent. No major shifts in serotype distribution were observed. Long-term surveillance data from Lombardy underscore the dominance of vaccine-targeted serotypes, ongoing circulation of resilient clones, and post-pandemic epidemiological shifts. These findings support continuous surveillance and inform vaccine strategy adjustments at the regional and national levels.

## 1. Introduction

Bacterial meningitis is related to high morbidity and mortality worldwide. Recently, an overall fatality ratio of 18% has been reported, which, if left untreated, could reach a mortality rate of up to 50% [1,2]. The incidence rate ranges from 0.9/100,000 population per year in high-income countries to 80/100,000 per year in low-income countries [3].

The primary pathogens responsible for bacterial meningitis are *Streptococcus pneumoniae* (SP), *Haemophilus influenzae* (HI) and *Neisseria meningitidis* (NM), each exhibiting unique serotype or serogroup variations that affect both the impact of the disease and the effectiveness of vaccines.

Specifically, SP is classified into more than 100 serotypes [4,5]. HI can be distinguished into six serotypes based on their unique polysaccharide capsule (a–f) [6]. NM is classified into 13 serogroups based on the polysaccharide capsule (A, B, C, D, 29-E, H, I, K, L, W-135, X, Y, and Z), with six (A, B, C, W-135, X, and Y—also known as MenA, MenB, MenC, MenW, MenX and MenY) responsible for most of the invasive meningococcal disease [7,8]. Non-encapsulated strains also exist in all three species and are termed non-typeable (for HI and SP) or non-groupable (NM) [9,10,11].

Widespread use of conjugate vaccines has significantly changed the epidemiology of bacterial meningitis, leading to a reduced incidence and a shift in serotype distribution.

In Italy, vaccination against HI, NM, and SP is not mandatory. The vaccination calendar for meningococcal disease, caused by NM, includes three types of vaccine: the conjugate vaccine against serogroup C, the tetravalent conjugate vaccine that protects against serogroups A, C, W135, Y and the conjugate vaccine against serogroup B. The Lombardy Region, with approximately 10 million inhabitants, offers the tetravalent vaccination to all individuals aged 1–18 years, while the MenB vaccine is recommended to all newborns, starting with the 2017 birth cohort [12]. For HI, the available vaccination is against serotype b and is administered from 2 months of age. Finally, for pneumococcal disease, caused by SP, there are two types of vaccine: the conjugate vaccine, of which there are different formulations containing a conjugate vaccine (with varying serotype coverage) and a 23-valent polysaccharide vaccine (PPV23) that can be used from 2 years of age and in adults [12]. However, variations in national vaccination schedules, emerging serotype replacement, and under-vaccinated subpopulations have sustained residual disease and underscore the importance of robust regional surveillance systems [13,14].

In the United States, the annual incidence declined from 2/100,000 population in 1998–1999 to 1.38/100,000 population in 2006–2007, while the mean age of patients increased from 30.3 years in 1998–1999 to 41.9 years in 2006–2007 [15]. Regarding Europe, a recent study from the Netherlands shows that the incidence of bacterial meningitis decreased from 6.37/100,000 population in 1989–1993 to 1.58/100,000 population per year in 2014–2019. This decrease was most pronounced in preschool and school-age children [13].

The National Institute of Health in Italy coordinates a surveillance system dedicated to invasive bacterial diseases by NM, SP and HI. Moreover, cases of invasive disease with microbiological confirmation are reported by the regions/autonomous provinces in the platform of invasive bacterial diseases (MaBI) [12]. In 2023, the incidence of meningococcal invasive disease cases in Italy was 0.14 cases/100,000 inhabitants; 3.02/100,000 for SP and 0.53/100,000 for HI cases [16]. Although national data indicate a low incidence of invasive disease, detailed regional trends, especially at the serotype level, remain insufficient, as there is great interregional variability in reporting with a decreasing gradient from North to South [16].

To address this gap, the Regional Reference Centre for Bacterial Invasive Diseases at Fondazione IRCCS Ca’ Granda Policlinico has conducted systematic monitoring of bacterial meningitis cases in Lombardy, the most populous Italian region, over the past decade. This study aims to describe trends in pathogen distribution and serogroup/serotype prevalence from 2014 to 2024, providing insight into vaccine effectiveness and residual disease burden.

## 2. Materials and Methods

### 2.1. Specimen Collection, Storage and Transport

This retrospective study, conducted from 1 January 2014 to 31 December 2024, included data from the Regional Reference Centre for Bacterial Invasive Diseases at the Fondazione IRCCS Ca’ Granda Policlinico hospital in the city of Milan–Lombardy Region, Italy. The centre received SP, HI and NM isolates obtained from cerebrospinal fluid (CSF) samples of patients diagnosed with meningitis and collected across 40 hospitals in the Lombardy Region. The viable bacterial isolates were delivered from each clinical centre at room temperature on plates or in transport medium buffers. Each sample was accompanied by a data sheet detailing the admission date and any administered antibiotic therapy. This study includes exclusively bacterial isolates referred to the Regional Reference Centre for Bacterial Invasive Diseases, which oversees laboratory surveillance for *S. pneumoniae* (SP), *N. meningitidis* (NM), and *H. influenzae* (HI). Other bacterial causes of meningitis are not systematically submitted to the centre and were not included in this analysis.

### 2.2. Detecting and Serotyping Pathogens

**Figure 1:**
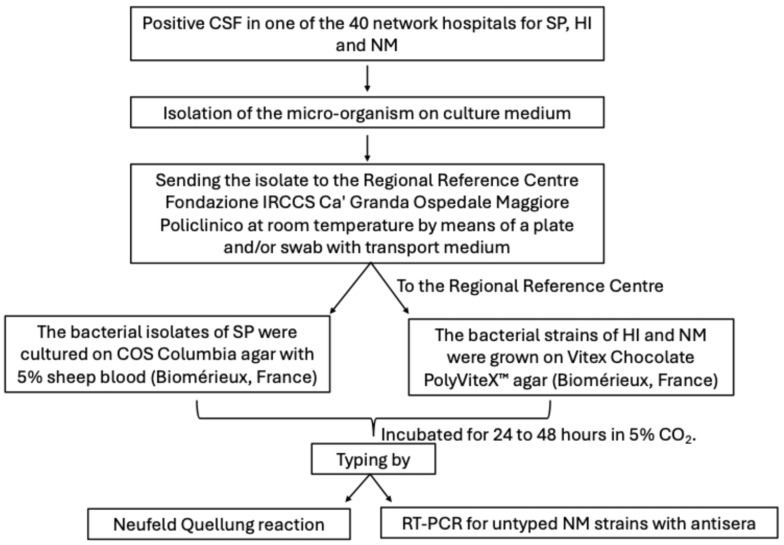
Operational procedure on surveillance of invasive bacterial diseases by *Streptococcus pneumoniae*, *Haemophilus influenzae* and *Neisseria meningitidis*.

The bacterial isolates of SP were cultured on COS Columbia agar with 5% sheep blood (Biomérieux, Marcy-l’Étoile, France). The bacterial strains of HI and NM were grown on Vitex Chocolate PolyViteX™ agar (Biomérieux, Marcy-l’Étoile, France). The cultures were incubated for 24 to 48 h in 5% CO_2_. Once the purity of the culture was verified, serotyping was performed the day after plating. The analysis was performed using the Neufeld Quellung reaction with commercial antisera for SP (ImmuLex^TM^ Pneumotest-Latex, SSI Diagnostica A/S, Hillerød, Denmark), HI (BD Difco^™^ H. Influenzae Antiserum, BD, Franklin Lakes, NJ, USA) and NM (Remel Agglutinating serum *Neisseria meningitidis* Group, Remel Microbiology Products – Thermo Fisher Scientific, Waltham, MA, USA), respectively. NM strains untyped by antisera underwent molecular characterisation performed using Real-time PCR (RT-PCR) (Sansure Biotech *Neisseria meningitidis* A, B, C Nucleic Acid Diagnostic Kit and Sansure Biotech *Neisseria meningitidis* W, X, Y Nucleic Acid Diagnostic Kit, Sansure Biotech Inc., Changsha, China). DNA was previously extracted by diluting the colonies in 200 µL of RNase-free water, followed by passage in thermoblock at 95 °C for 10 min and centrifugation at 14 rpm for 5 min. Reaction mixtures were prepared by obtaining a final volume of 22 µL, consisting of 2 µL of extracted DNA, 19 µL of Master Mix (sacB/synD/snyE PCR Mix, Sansure Biotech Inc., Changsha, China) and 2 µL of enzyme (sacB/synD/snyE PCR Enzyme Mix, Sansure Biotech Inc., Changsha, China). The thermal profile conditions were 1 cycle at 50 °C for 2 min, followed by 1 cycle at 95 °C for 5 min, 45 cycles at 95 °C for 45 s and finally 25 °C for 10 s. Each run included both positive and negative controls. The operational procedure described for surveillance of invasive bacterial diseases by SP, NM and HI is shown in Figure 1.

### 2.3. Data Analysis

Data were analysed by aetiological agent, serogroup, age group (infants: <1 year; young children: 1–5 years; older children: 6–17 years; adults: 18–64 years; elderly: ≥65 years) and year of diagnosis. Epidemiological and laboratory data were analysed using the Jonckheere–Terpstra and Chi-square tests, where appropriate. Statistical significance was set at *p* < 0.05.

## 3. Results

### 3.1. Epidemiological Characteristics

During the study period, 731 CSF isolates positive for bacterial meningitis. Pathogens were received at the Microbiology and Virology Unit of IRCCS Fondazione Ca’ Granda Policlinico. The median number of samples received yearly was 66 (50–80), with the lowest observed in 2020 (30/731, 4.1%) and 2021 (26/731, 3.5%). The highest number of samples was received in 2017 (108/731, 14.8%), followed by 2016 (101/731, 13.8%). The fluctuations in annual case counts did not constitute a statistically significant decreasing trend (Table 1). The median (interquartile range, IQR) age of positive cases was 60 (41–71) years. No difference in the median age was observed across years (*p* = 0.846). The median ages of infants, young and older children, adults and the elderly were 0.5 (0.3–0.6) months, 3 (1–4), 12 (9–15), 50 (40–58) and 73 (68–78) years, respectively.

Cases were distributed across age groups as follows: 3.6% (26/731) in infants, 4.7% (34/731) in young children, 4.4% (32/731) in older children, 45.6% (333/731) in adults, 41.9% (306/731) in elderly. The percentage of positive cases in infants showed an erratic trend over the years, with a minimum of 0% in 2014–2020–2021–2024 and a maximum of 9.6% (5/52) in 2023 (*p* = 0.011). A similar trend was observed for positive cases in older children, with a prevalence that varied from a minimum of 0% in 2021 and 2022 to a maximum of 11.3% (7/62) in 2014 (*p* = 0.009). No difference was found in the percentage of positive cases in young children, adults and elderly against time (Table 1).

Looking at the distribution of etiological agents of bacterial meningitidis against age, we found that SP and HI were most frequently detected among adults and elderly with respect to infants and young and older children (SP: 259 [45.2%] and 270 [47.1%] in adults and elderly, respectively; HI: 25 [39.5%] in adults and elderly category). On the other hand, NM was more common among adults (51.6%, 49/95), followed by older (17.0%, 16/95) and young children (14.7%, 14/95) (Table 2).

### 3.2. SP, NM and HI Trends

Of the 731 total positive samples, SP was the most prevalent, accounting for 78.4% (573/731), followed by NM at 13.0% (95/731) and HI at 8.6% (63/731) (Supplementary File S1).

From 2014 to 2024, analysis of pathogen distribution revealed significant temporal variation for SP (*p* = 0.046) and NM (*p* = 0.023), whereas HI did not show significant fluctuations (*p* = 0.316).

SP consistently emerged as the predominant pathogen, typically comprising 65.0–92.3% of annual cases.

Although the proportion of SP among total meningitis cases peaked in 2023 (92.3%, 48/52), this increase did not reflect a corresponding rise in absolute SP cases. Case counts remained relatively stable in recent years, with 52 (65.0%) cases in 2019 and 49 (74.2%) in 2024.

*H. influenzae* exhibited a more variable trend over time. Starting at 4.8% (3/62) in 2014, its prevalence varied over the years, peaking at 15.4% (4/26) in 2021, with a low total cases count that year, dropping to 5.8% (3/52) in 2023, and rising again to 13.6% (9/66) in 2024. In absolute terms, the number of HI isolates ranged from 2 to 9 cases per year.

NM showed an overall declining trend. Initially representing 19.4% (12/62) of cases in 2014, the proportion declined to 11.9% (12/101) in 2016 and 9.3% (10/108) in 2017. A temporary spike occurred in 2019 (23.8%, 19/80), but this was followed by a sharp decline to 1.9% (1/52) in 2023. In 2024, NM rebounded slightly to 12.1% (8/66). The total number of NM cases peaked in 2019 with 19 cases and declined thereafter, particularly during the COVID-19 pandemic and subsequent years (2020–2022), when only 3–4 cases were reported annually (Table 3).

### 3.3. Distribution of Serotypes of SP Isolates

**Figure 2:**
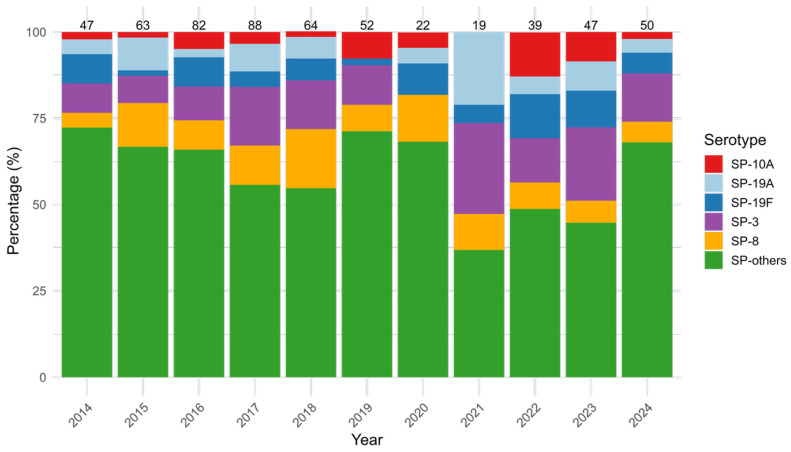
Yearly distribution (%) of the five most prevalent *Streptococcus pneumoniae* (SP) serotypes (SP-3, SP-8, SP-19A, SP-19F, SP-10A) and others (SP-others), from 2014 to 2024. Bars represent the percentage of each serogroup among annual cases, with total case counts displayed above each column.

To assess temporal trends in the distribution of SP serogroups from 2014 to 2024, we evaluated both annual prevalence and year-to-year variability. SP-3 was the most common SP identified (12.9%, 74/573), and it was most frequently found in adults (48.6%, 36/74) and elderly (45.9% 34/74), while it was rarely found in infants (one case), young children (one case) and older children (two cases). SP-3 did not show significant changes over time (*p* = 0.187), ranging from 0.0% (0/22) in 2020 to 26.3% (5/19) in 2021, with an additional peak in 2023 (21.3%, 10/47). SP-8 followed with an overall prevalence of 9.8% (56/573) and did not change significantly over time (*p* = 0.544), even if it increased from 4.3% (2/47) in 2014 to a peak of 17.2% (11/64) in 2018. SP-19A (*p* = 0.093), SP-19F (*p* = 0.441), and SP-10A (*p* = 0.142) showed no statistically significant year-to-year changes as well, suggesting stable distribution patterns. Other SP types, with single prevalence values ranging from 0.1% (1/573) to 4.0% (23/573), were grouped as SP-others, accounting for 60.5% (347/573) of all SP cases, and demonstrated significant variation over time (*p* = 0.013), with annual proportions ranging from 72.3% (34/47) in 2014 to 71.2% (37/52) in 2019 and 68.0% (34/50) in 2024 (Figure 2).

### 3.4. Distribution of Serotypes of HI Isolates

**Figure 3:**
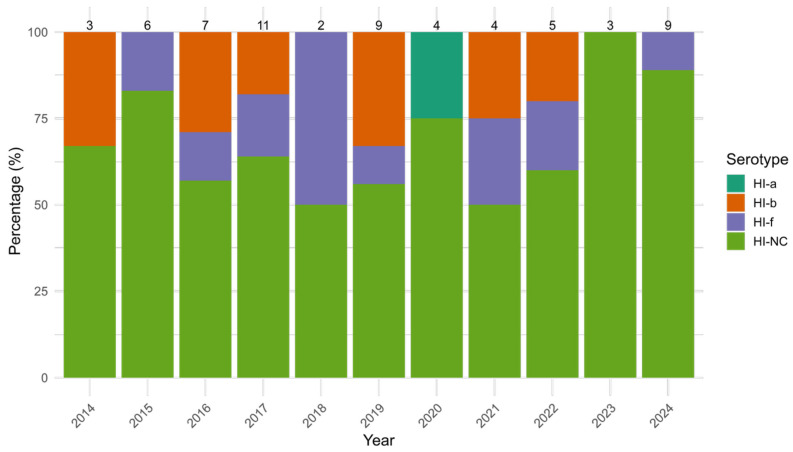
Yearly distribution (%) of *Haemophilus influenzae* (HI) serotypes (HI-a, HI-b, HI-f and non-determinable serotype HI-NC) from 2014 to 2024. Bars represent the percentage of each serogroup among annual cases, with total case counts displayed above each column.

Among HI isolates, HI-NC was the most common serotype, accounting for 68.3% (43/63) of HI cases. It showed the highest prevalence among adults (51.1%, 22/43) and the elderly (37.2%, 16/43), with no cases observed in older children. Its detection rate ranged from 50.0% (1/2) in 2018 to 100% (3/3) in 2023, with non-significant variation over time (*p* = 0.803). HI-f had an overall prevalence of 14.3% (9/63), ranging from 0.0% (0/3) in 2014 to 50.0% (1/2) in 2018; again, non-significant variation was detected (*p* = 0.918). HI-b represented 15.9% of all HI cases, varying between 0.0% (0/6) in 2015 and 33.3% in 2014 (1/3) and 2019 (3/9), without statistical significance (*p* = 0.583). HI-a, the least common strain, accounted for only 1.6% of HI isolates, only being detected in 2020 in 1 out 4 HI (25.0%) (Figure 3). No HI-e was detected.

### 3.5. Distribution of Serogroups of NM Isolates

**Figure 4:**
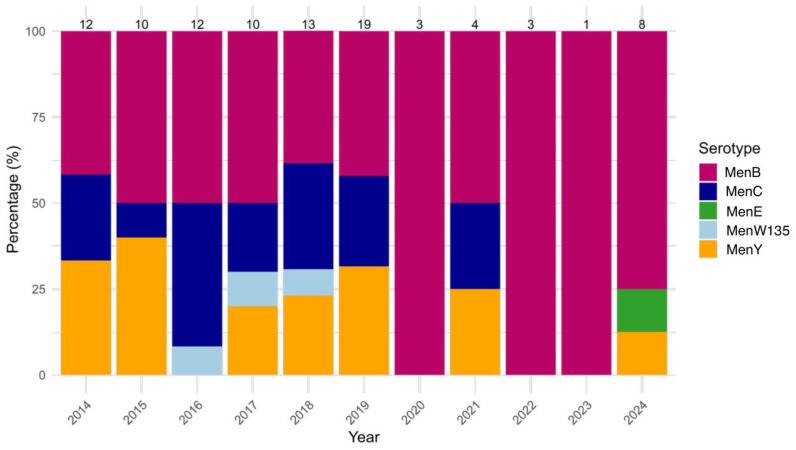
Yearly distribution (%) of *Neisseria meningitidis* (NM) serogroups (MenB, MenC, MenE, MenW135, MenY) from 2014 to 2024. Bars represent the percentage of each serogroup among annual cases, with total case counts displayed above each column.

Regarding NM, MenB was the most prevalent serogroup, accounting for 51.6% (49/95) of all NM cases observed during the study period, followed by MenC and MenY, with each reported in 22.1% of the cases (MenC: 21/95, MenY: 21/95). MenB cases were most found in adults (46.9%, 23/49), as well as MenC (57.1%, 12/21) and MenY (100%, 3/3). Other MenB cases were frequently observed among young children (20.4%, 10/49). The annual prevalence of MenB varied from 38.5% (5/13) in 2018 to 100% in 2020 (3/3), 2022 (3/3) and 2023 (1/1); however, this fluctuation was not statistically significant (*p* = 0.404). The prevalence of MenC also showed no significant variation, ranging from 41.7% (5/12) in 2016 to 0.0% (0/8) in 2024 (*p* = 0.566). Similarly, MenY exhibited year-to-year fluctuation (*p* = 0.476), with its prevalence ranging from 33.3% (4/12) in 2014 to 12.5% (1/8) in 2024. In contrast, MenE and MenW135 remained rare throughout the study period, with an overall observation rate equal to 1.1% (1/95) for MenE, and 3.2% (3/95) for MenW135. Neither of these serogroups showed statistically significant changes (MenE: *p* = 0.358, MenW135: *p* = 0.862) (Figure 4).

## 4. Discussion

Our study describes the serogroup and serotype distribution of the three main bacterial pathogens associated with meningitidis in Lombardy over the past eleven years. The analysis was made possible through an ongoing Surveillance Protocol for invasive diseases caused by SP, NM, and HI, jointly established by the IRCCS Fondazione Ca’ Granda Policlinico and the Lombardy Region, which also designated the IRCCS as the regional Reference Center. As part of the surveillance system, forty hospitals sent to the IRCCS all bacterial strains isolated from the CSF of patients diagnosed with meningitis. These strains were then serotyped using agglutination and molecular typing methods.

From 2014 to 2024, 731 samples were collected: 78% were SP, 13% NM, and 9% HI. Pathogen distribution revealed significant temporal variation for SP and NM, while HI showed no significant fluctuations. SP remained the predominant pathogen, HI displayed irregular trends, and NM followed a regular but declining pattern, with a temporal increase in 2019.

During our study period, SP and HI were predominantly reported in the elderly (47.1% and 39.5%, respectively) and in adults (45.2% and 39.5%, respectively). The SP data are in line with the data provided by the ECDC [17], where the highest incidence (12.6 confirmed cases per 100,000 inhabitants) is observed in a population of more than 65 years. Circulation of HI in Europe has instead the highest incidence among individuals of less than one year of age (specifically 7.0 cases per 100,000 population for male infants and 4.5 for female infants) [18], while in our data the highest number of HI cases was observed in persons aged >18 years. Similar results were observed for NM cases that in our data were more frequently diagnosticated in adults with respect to infants and young children (51.6% vs. 5.2% vs. 14.7%), unlike ECDC data, which showed the NM highest prevalence in infants, followed by young children and adolescents [19]. It should be noted that ECDC data are expressed in terms of incidence, considering the population at risk in each age group, whereas our data refer to the absolute number of diagnosed cases, without normalization for the size of the reference population. This methodological difference may affect the interpretation of the results and help explain the apparent discrepancies compared to European data.

While the number of HI cases remained similar in the years 2020–2021, a marked decline in SP and NM cases was observed, coinciding with the COVID-19 pandemic and the implementation of public health measures such as social distancing and mask mandates. These trends are consistent with findings from other countries. For example, a Brazilian retrospective study showed decreased hospitalizations for meningitis in all but two States during 2020 versus the 2015–2019 average [20]. Similarly, a study from United States evaluating the period from March to December 2020 reported a 58% decline in SP infections [21]. Comparable decreases were reported in Uruguay and the Netherlands [22,23]. The unchanged number of HI cases in 2020–2021 reflects a similar condition in France [24]. This is due to a decrease in HI-NC with an increase in capsulated serotypes such as serotype a and b. This phenomenon could be attributed to increased difficulty in accessing vaccination programs during the pandemic period and the partial relaxation of restrictions in the second half of the pandemic that favored transmission within small networks [24].

Among EU/EEA countries, the most common serotypes in 2022, in order of decreasing frequency, were 3, 8, 19A, 22F, 6C, 23B, 9N, 4, 23A, 11A and 15A. Compared with 2018, there was an increase in serotypes 3, 19A and 6C in 2022, by 33%, 40% and 27%, respectively. During the same period, a decrease was observed in serotypes SP-22F and SP-9N, by 17% and 31%, respectively [17]. In contrast to the ECDC data, our results show the emergence of serotype 10A in 2022 and the increase in serotype 3. SP-10A, not historically dominant in Europe, showed an upward trend in Lombardy, consistently with a South Korean study reporting SP-10A as the most common non-vaccine serotype (23.8%) [25,26]. The increase in this serotype could be linked to the spread of new clones. These observations corroborate the hypothesis that granular surveillance regarding SP types, like the ongoing program in the Lombardy Region, is essential to adjust vaccination strategies based on the molecular epidemiology of microorganisms.

SP-3 had the highest prevalence in our population and was mostly found in adults and the elderly, highlighting the importance of polysaccharide-conjugated vaccines (PCVs), that includes SP-3, offered for free to individuals >65 years old in Lombardy [12]. This was followed in frequency by serotype 8. Despite its inclusion in PCV-13 and PCV-23, serotype 3 remains a significant cause of invasive pneumococcal disease (IPD) globally [27,28]. regarding addition, in Portugal, a significant increase in IPD, frequently caused by SP-3, occurred in 2018–2023 [29]. The prevalence of serotype 3 also shows substantial geographic variation and is known for its increased propensity for genetic recombination and segregation compared to other serotypes [30,31]. While clonal complex 180 (CC180) is the most dominant SP-3 strain worldwide with its three main sublineages (Iα, Iβ, and II), in the United States, a global increase in Clade II with respect to Clade I was observed following the introduction of the PCV13 vaccine. Similarly, Clade II emerged as lineage in England and Wales [32,33,34]. This clade is characterized by a higher prevalence of antimicrobial resistance (AMR), greater diversity of surface protein antigens, and a higher rate of recombination [30]. These features suggest that Clade II may have a greater potential for vaccine escape compared to Clade I. Given these trends, it would be valuable to investigate the circulating clade distribution of serotype 3 in Italy, and to explore potential correlations with patient age and temporal patterns.

The continuous detection of SP serotype 19A and 19F, covered by existing vaccines including PCV7 and PCV13, is in line with other published literature [35,36]. An Irish study highlighted the persistent circulation of serotype 19A, which may be driven by the dissemination of highly antibiotic-resistant clones, such as GPSC1-CC320. Additionally, relatively high antibiotic prescription rates, both in Ireland and in Polonia, are believed to have further facilitated the spread of this clone [35,37,38]. A 2018 United States study reported a rise in 19F colonization associated with the emergence of a novel clone, ST9074 [36]. This clone is thought to reduce antibody avidity against the pneumococcal capsule and may exhibit resistance to complement-mediated clearance, through mechanisms likely similar to other invasive strains [28].

Regarding SP-8, its continuous persistence in Lombardy over time may be suggestive of the circulation of specific clones, as suggested by the rise of Netherlands 8-ST53 in Denmark [39].

Further molecular investigations, including genomic sequencing, are warranted to elucidate clonal dynamics and vaccine pressure effects.

The observed trend in NM cases aligns with international findings that describe a decline in NM incidence followed the pandemic restrictions, with a subsequent increase after their removal [40,41]. A population-based study from Greece reported a 70% decrease in NM incidence among children aged 0–14 years during 2020–2021, compared to the 2014–2019 period [42]. Our data show a spike in NM cases in 2019, potentially attributed to a localized outbreak in the Bergamo area of the Lombardy Region, between December 2019 and January 2020 [43]. In contrast, Morocco reported an increase in pediatric meningitis cases during the pandemic compared to the pre-pandemic period, possibly due to healthcare disruptions and delays in care-seeking associated with COVID-19 waves [44].

In our setting, MenB remained the most frequent serogroup (52%), followed by MenC and MenY (each 22%). This is consistent with European epidemiology, where serogroups B, W, and Y are predominant [19,45]. Despite this, in the last ECDC report [17], between 2018 and 2022, there was an overall increase in the notification rate of NM serogroups B, W and Y in 2022 compared to 2021, while the incidence of serogroup C has been decreasing and remained at the same low level as 2021. Again, the ECDC report is consistent with our data, which described a decrease in MenC cases, from 42% in 2016 to 0% in 2024, and an increase in MenY, in line with observations in other parts of Europe [19,45]. This may suggest a trend in MenC disappearing from Europe and the emergence of other serogroups (but mostly MenY and MenW), as suggested by other reports [45,46]. Among other serogroups, MenW remained rare in our region despite increases elsewhere in Europe. In the Netherlands, MenW incidence increased tenfold between 2015 and 2017, driven by a UK-linked clone spreading across multiple countries [46]. Notably, *Neisseria meningitidis* serogroup E (MenE), which has only been rarely detected in Europe since 1993 and reported in a few isolated cases in Australia in 2017 [47,48], was identified for the first time in Lombardy in 2024. This finding may confirm a potential shift in *N. meningitidis* epidemiology from MenC to other serotypes.

Among HI isolates, HI-NC was the most common, representing 68.2% of cases. Among capsulated types, HI-b (16%) remained the most common, followed by HI-f (14%). This aligns with global and European trends; indeed, between 2007 and 2014, 78% of HI invasive disease in Europe was caused by non-typeable strains [49,50,51]. The ECDC report in 2022 reported 2307 cases of invasive HI disease with known serotypes (58%) [18]. Among these, non-capsulated (non-typeable) strains were the most common, accounting for 1685 cases (73%) [18]. The high prevalence of non-typeable strains may be linked to their pronounced genetic variability, which could facilitate evasion of host immune responses [52,53]. Serotype b (Hib) was the next most frequent, with 211 cases (9.1%). The continued presence of Hib in Europe remains a concern despite widespread vaccination. In Lombardy, vaccination against Hib is administered starting at 2 months of age [12]. Nevertheless, Hib has remained the most detected capsulated strain in the region, with its prevalence fluctuating from 0.0% in 2015 to 33.3% in both 2014 and 2019. Notably, no Hib cases were reported in the most recent two years. The Netherlands reported increased HI-b incidence during 2020–2021, with 40% of cases affecting children under 5 years of age [54]. In France, HI-b also surged, with 66% of all cases occurring in 2019 [55]. Regarding the other capsulated strains, HI-a (1.6%) emerged in our setting, corroborating the hypothesis of emergence in Italy [56]. Increasing Hi-a cases were reported in Europe and America. For example, in Norway, HI-a accounted for 22% of invasive disease cases from 2018 to 2021. In France, HI-a peaked in 2020, while in Argentina, its prevalence increased from 12% to 15% between 2011 and 2019 [6,57,58,59,60,61].

This study has several limitations. It is based on passive laboratory surveillance, which may underestimate the actual disease burden due to missed or unreported cases and other important causes of bacterial meningitis, including pathogens such as *S. agalactiae*, *E. coli*, and *L. monocytogenes*, are not systematically included. Additionally, we were unable to determine the total number of meningitis cases diagnosed regionally, as only culture-confirmed cases submitted to the center were included. These constraints may underestimate the overall disease burden and limit generalizability to all meningitis etiologies. Clinical data, including vaccination status and patient outcomes, were not collected, limiting assessment of vaccine effectiveness or disease severity. Moreover, molecular typing or genomic analysis was not performed, preventing deeper insight into clonal dynamics or antimicrobial resistance. Despite these limitations, the study’s strength lies in its comprehensive, region-wide coverage and standardized laboratory procedures.

## 5. Conclusions

Our findings provide a comprehensive epidemiological profile of bacterial meningitis in Lombardy over more than a decade, highlighting the continued dominance of vaccine-included serotypes such as SP-3, the high prevalence of HI-NC, and the evolving serogroup distribution of NM in the region. A temporary reduction in cases was observed during the COVID-19 pandemic in Lombardy and, to a greater extent, in Italy, confirming that population-wide behavioral changes, such as social distancing and mask-wearing, had an impact on the transmission of other pathogens [62,63]. These findings underscore the critical role of sustained laboratory surveillance, vaccine program evaluation and the integration of genomic tools to track emerging clones and inform public health strategies.

## Figures and Tables

**Table 1:** Epidemiological characteristics of the 731 samples of meningitis-positive cases. Abbreviation: *n* = number; IQR = interquartile range.

Sample Characteristics	Overall	Years											*p*-Value
		2014	2015	2016	2017	2018	2019	2020	2021	2022	2023	2024	
*N* (%)	731 (10)	62 (8.4)	79 (10.8)	101 (13.8)	108 (14.7)	79 (10.8)	80 (10.9)	30 (4.1)	26 (3.5)	48 (6.5)	52 (7.1)	66 (9.0)	0.137
Age in years, median (IQR)	60 (41–71)	60 (36–71)	60 (47–71)	62 (43–68)	62 (38–73)	57 (44–73)	57 (31–68)	63 (32–74)	56 (50–65)	59 (46–71)	57 (40–71)	62 (41–72)	0.846
Infants, *n* (%)	26 (3.6)	0 (0.0)	3 (3.8)	1 (0.9)	8 (7.4)	1 (1.2)	5 (6.3)	0 (0.0)	0 (0.0)	3 (6.2)	5 (9.6)	0 (0.0)	0.011
Young children, *n* (%)	34 (4.7)	6 (9.7)	2 (2.5)	6 (5.9)	5 (4.7)	3 (3.8)	2 (2.5)	2 (6.6)	1 (3.8)	3 (6.2)	1 (1.9)	3 (4.5)	0.71
Older children, *n* (%)	32 (4.4)	7 (11.3)	1 (1.3)	3 (3.0)	2 (1.9)	1 (1.3)	7 (8.8)	2 (6.6)	0 (0)	0 (0)	4 (7.7)	5 (7.6)	0.009
Adults, *n* (%)	333 (45.6)	21 (34)	39 (49.4)	48 (48.6)	43 (39.8)	42 (53.2)	37 (46.2)	12 (40.0)	18 (69.2)	22 (45.9)	24 (46.2)	27 (40.9)	0.157
Elderly, *n* (%)	306 (41.9)	28 (45)	34 (43.0)	43 (41.6)	50 (46.2)	32 (40.5)	29 (36.2)	14 (46.7)	7 (27.0)	20 (41.7)	18 (34.6)	31 (47.0)	0.744

**Table 2:** Distribution of *Streptococcus pneumoniae*, *Haemophilus influenzae* and *Neisseria meningitidis* among infants, young and older children, adults and elderly.

Pathogens	Infants, *n* (%)	Young Children, *n* (%)	Older Children, *n* (%)	Adults, *n* (%)	Elderly, *n* (%)	*p*-Value
*S. pneumoniae*, *n* = 573	13 (2.3)	15 (2.6)	16 (2.8)	259 (45.2)	270 (47.1)	<0.001
*H. influenzae*, *n* = 63	8 (13.0)	5 (8.0)	0 (0.0)	25 (39.5)	25 (39.5)	<0.001
*N. meningitidis*, *n* = 95	5 (5.2)	14 (14.7)	16 (17.0)	49 (51.6)	11 (11.5)	<0.001

**Table 3:** Prevalence (%) of *Streptococcus pneumoniae* (SP), *Neisseria meningitidis* (NM) and *Haemophilus influenzae* (HI) for each study year, 2014–2024.

Pathogens	Years											*p*-Value
	2014	2015	2016	2017	2018	2019	2020	2021	2022	2023	2024	
*S. pneumoniae*, *n* (%)	47 (75.8)	63 (79.7)	82 (81.2)	87 (80.5)	64 (81.0)	52 (65.0)	23 (76.7)	18 (69.2)	40 (83.3)	48 (92.3)	49 (74.2)	0.046
*H. influenzae*, *n* (%)	3 (4.8)	6 (7.6)	7 (7.0)	11 (10.2)	2 (2.5)	9 (11.2)	4 (13.3)	4 (15.4)	5 (10.4)	3 (5.8)	9 (13.7)	0.316
*N. meningitidis*, *n* (%)	12 (19.4)	10 (12.7)	12 (11.8)	10 (9.3)	13 (16.5)	19 (23.8)	3 (10.0)	4 (15.4)	3 (6.3)	1 (1.9)	8 (12.1)	0.023

## Data Availability

The original contributions presented in this study are included in the article as aggregated data. Further inquiries can be directed to the corresponding author.

## Supplementary Materials

The following supporting information can be downloaded at https://www.mdpi.com/article/10.3390/microorganisms13081733/s1. Supplementary File S1: Prevalence of all *Streptococcus pneumoniae*, *Haemophilus influenzae* and *Neisseria meningitidis* types detected in the study period.

## Abbreviations

The following abbreviations are used in this manuscript:

SP	* Streptococcus pneumoniae *
HI	* Haemophilus influenzae *
NM	* Neisseria meningitidis *
CSF	Cerebrospinal fluid
PPV	Polysaccharide vaccine
